# Novel inflammatory markers for incident pre-diabetes and type 2 diabetes: the Rotterdam Study

**DOI:** 10.1007/s10654-017-0236-0

**Published:** 2017-03-03

**Authors:** Adela Brahimaj, Symen Ligthart, Mohsen Ghanbari, Mohammad Arfan Ikram, Albert Hofman, Oscar H. Franco, Maryam Kavousi, Abbas Dehghan

**Affiliations:** 1 Department of Epidemiology, Erasmus University Medical Center, P.O. Box 2040, 3000 CA Rotterdam, The Netherlands; 2 Department of Neurology, Erasmus University Medical Center, Rotterdam, The Netherlands; 3 Department of Radiology, Erasmus University Medical Center, Rotterdam, The Netherlands; 4 Harvard School of Public Health, Boston, MA USA

**Keywords:** Inflammatory markers, Phase-specific, Pre-diabetes, Type 2 diabetes, Insulin therapy, Novel, IL13, IL17, EN-RAGE

## Abstract

The immune response involved in each phase of type 2 diabetes (T2D) development might be different. We aimed to identify novel inflammatory markers that predict progression from normoglycemia to pre-diabetes, incident T2D and insulin therapy. We used plasma levels of 26 inflammatory markers in 971 subjects from the Rotterdam Study. Among them 17 are novel and 9 previously studied. Cox regression models were built to perform survival analysis. Main Outcome Measures: During a follow-up of up to 14.7 years (between April 1, 1997, and Jan 1, 2012) 139 cases of pre-diabetes, 110 cases of T2D and 26 cases of insulin initiation were identified. In age and sex adjusted Cox models, IL13 (HR = 0.78), EN-RAGE (1.30), CFH (1.24), IL18 (1.22) and CRP (1.32) were associated with incident pre-diabetes. IL13 (0.62), IL17 (0.75), EN-RAGE (1.25), complement 3 (1.44), IL18 (1.35), TNFRII (1.27), IL1ra (1.24) and CRP (1.64) were associated with incident T2D. In multivariate models, IL13 (0.77), EN-RAGE (1.23) and CRP (1.26) remained associated with pre-diabetes. IL13 (0.67), IL17 (0.76) and CRP (1.32) remained associated with T2D. IL13 (0.55) was the only marker associated with initiation of insulin therapy in diabetics. Various inflammatory markers are associated with progression from normoglycemia to pre-diabetes (IL13, EN-RAGE, CRP), T2D (IL13, IL17, CRP) or insulin therapy start (IL13). Among them, EN-RAGE is a novel inflammatory marker for pre-diabetes, IL17 for incident T2D and IL13 for pre-diabetes, incident T2D and insulin therapy start.

**Electronic supplementary material:**

The online version of this article (doi:10.1007/s10654-017-0236-0) contains supplementary material, which is available to authorized users.

## Introduction

There is increasing evidence that inflammation plays a role in the development of type 2 diabetes mellitus (DM) [1–3]. In this context, the identification of novel inflammatory markers associated with the risk of type 2 DM will shed light on the pathophysiology of the disease and might also help clinicians to target individuals at highest risk [4, 5]. So far, a limited number of inflammatory markers have been investigated. Previous studies reported inflammatory markers including C-reactive protein (CRP), interleukin 6 (IL6) and adiponectin to associate with the risk of type 2 DM [6–11]. These studies merely investigated inflammatory markers that predict the conversion from normoglycemia to type 2 DM.

Healthy individuals are thought to experience a pre-diabetes phase before developing type 2 DM. Pre-diabetes is the presence of blood glucose levels higher than normal, but not yet high enough to be classified as diabetes [12]. Moreover, type 2 DM could further deteriorate to a stage, where glucose control is only possible by insulin therapy [12, 13]. Progression from normoglycemia to pre-diabetes is thought to be driven by insulin resistance, while progression to type 2 DM and need for insulin therapy is further affected by beta cell dysfunction [14–16]. Therefore, the immune response involved in each of these phases might be different [17].

We hypothesized that inflammatory markers are phase-specific for conversion from normoglycemia to pre-diabetes, diabetes and need for insulin therapy. We agnostically studied the association of a set of inflammatory markers with progression from normoglycemia to pre-diabetes, type 2 DM and finally to insulin therapy.

## Materials and methods

### Study population

The Rotterdam Study is a prospective population-based cohort study in Ommoord, a district of Rotterdam, the Netherlands. The design of the Rotterdam Study has been described in more detail elsewhere [18]. Briefly, in 1989 all residents within the well-defined study area aged 55 years or older were invited to participate of whom 78% (7983 out of 10,275) agreed. There were no other eligibility criteria to enter the Rotterdam Study except minimum age and residency are based on ZIP code. The first examination took place from 1990 to 1993, after which follow-up examinations were conducted every 3–5 years. This study was based on data collected during the third visit (1997–1999). We used data from 971 individuals with available data on inflammatory markers, drawn as a random control sample in a case-cohort study of markers for dementia. The Rotterdam Study has been approved by the medical ethics committee according to the Population Screening Act: Rotterdam Study, executed by the Ministry of Health, Welfare and Sports of Netherlands. All participants in the present analysis provided written informed consent to participate and to obtain information from their treating physicians.

### Measurement of inflammatory markers

Fasting blood samples were collected at the research centre. Plasma was isolated and immediately put on ice and stored at −80 °C. Citrate plasma (200Ul) was sent in July 2008 to Rules-Based Medicine, Austin, Texas (www.myriadrbm.com). The samples were thawed at room temperature, vortexed, spun at 4000 RPM for 5 min for clarification and volume was removed for MAP analysis into a master microtiter plate. Using automated pipetting, an aliquot of each sample was introduced into one of the capture microsphere multiplexes of the Multi Analyte Profile. The mixture of sample and capture microspheres were thoroughly mixed and incubated at room temperature for 1 h. Multiplexed cocktails of biotinylated, reporter antibodies for each multiplex were then added robotically and after thorough mixing, were incubated for an additional hour at room temperature. Multiplexes were developed using an excess of streptavidin–phycoerythrin solution which was thoroughly mixed into each multiplex and incubated for 1 h at room temperature. The volume of each multiplexed reaction was reduced by vacuum filtration and the volume increased by dilution into matrix buffer for analysis. Analysis was performed in a Luminex 100 instrument and the resulting data stream was interpreted using proprietary data analysis software developed at Rules-Based Medicine (https://myriadrbm.com/scientific-media/quality-control-systems-white-paper/). For each multiplex, both calibrators and controls were included on each microtiter plate. 8-point calibrators were run in the first and last column of each plate and 3-level controls were included in duplicate. Testing results were determined first for the high, medium and low controls for each multiplex to ensure proper assay performance. Unknown values for each of the analytes localized in a specific multiplex were determined using 4 and 5 parameter, weighted and non-weighted curve fitting algorithms included in the data analysis package.

Fifty inflammatory markers were quantified using multiplex immunoassay on a custom designed human multi-analyte profile. The intra-assay variability was less than 4% and the inter assay variability was less than 13%. Markers with more than 60% completeness of measurements were selected for analysis (26 from 50) [19].

### Type 2 diabetes mellitus diagnosis

The participants were followed from the date of baseline center visit onwards. At baseline and during follow-up, cases of pre-diabetes and type 2 DM were ascertained through active follow-up using general practitioners’ records, hospital discharge letters and glucose measurements from Rotterdam Study visits which take place approximately every 4 years [20]. Diabetes, pre-diabetes and normoglycemia were defined according to the current WHO guidelines. Normoglycemia was defined as a fasting blood glucose level <6.0 mmol/L; pre-diabetes was defined as a fasting blood glucose between 6.0 and 7.0 mmol/L or a non-fasting blood glucose between 7.7 and 11.1 mmol/L (when fasting samples were unavailable); type 2 diabetes was defined as a fasting blood glucose ≥7.0 mmol/L, a non-fasting blood glucose ≥11.1 mmol/L (when fasting samples were unavailable), or the use of blood glucose lowering medication [20]. Information regarding the use of blood glucose lowering medication was derived from both structured home interviews and linkage to pharmacy dispensing records. At baseline, more than 95% of the Rotterdam Study population was covered by the pharmacies in the study area. All potential events of pre-diabetes and type 2 diabetes were independently adjudicated by two study physicians. In case of disagreement, consensus was sought with an endocrinologist. Follow-up data was complete until January 1st 2012, calculated as a separate variable for every outcome, taking in account the hierarchy of events as follows: pre-diabetes, type 2 diabetes, insulin therapy start [20].

### Covariates

Height and weight were measured with the participants standing without shoes and heavy outer garments. Body mass index (BMI) was calculated as weight divided by height squared (kg/m^2^). Waist circumference was measured at the level midway between the lower rib margin and the iliac crest with participants in standing position without heavy outer garments and with emptied pockets, breathing out gently. Blood pressure was measured at the right brachial artery with a random-zero sphygmomanometer with the participant in sitting position, and the mean of 2 consecutive measurements was used. Information on medication use, medical history and smoking behaviour was collected via computerized questionnaires during home visits. Smoking was classified as current versus non-current smokers. Participants were asked whether they were currently smoking cigarettes, cigars, or pipes. History of cardiovascular disease was defined as a history of coronary heart diseases (myocardial infarction, revascularization, coronary artery bypass graft surgery or percutaneous coronary intervention) and was verified from the medical records of the general practitioner. Alcohol intake was assessed in grams of ethanol per day. Insulin, glucose, total cholesterol (TC), high-density lipoprotein cholesterol (HDL-C), triglycerides (TG) were measured on the COBAS 8000 Modular Analyzer (Roche Diagnostics GmbH). The corresponding interassay coefficients of variations are the following: insulin <8%, glucose <1.4%, lipids <2.1%. HOMA-IR (the homeostatic model assessment to quantify insulin resistance) was calculated dividing the product of fasting glucose (in mmol/L) and fasting insulin (in mU/L) by 22.5. HOMA-B (the homeostatic model assessment of β-cell function) was calculated dividing the product of fasting insulin (in mU/L) and 20 by the difference of glucose (in mmol/L) with 3.5 [21].

### Statistical analyses

**Fig. 1:**
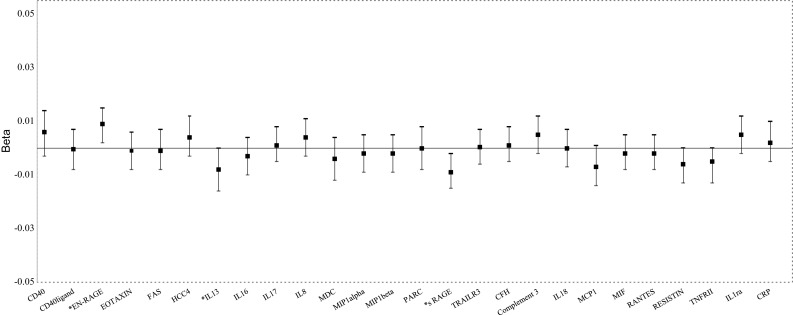
Associations of inflammatory markers with fasting glucose. CD40, cluster of differentiation 40; CD40 ligand, cluster of differentiation 40 ligand; EN-RAGE, Extracellular Newly identified Receptor for Advanced Glycation End-products binding protein; FAS, Fas Cell Surface Death Receptor; HCC4, Human CC chemokine-4; IL13, interleukin 13; IL16, interleukin 16; IL17, interleukin 17; IL8, interleukin 8; MDC, Monocyte Derived Chemokine; MIP1alpha, Macrophage Inflammatory Protein 1 alpha; MIP1beta, Macrophage Inflammatory Protein 1 beta; PARC, Pulmonary and Activation-Regulated Chemokine; sRage, Soluble Receptor of Advanced Glycation End-products; TRAILR3, Tumor Necrosis Factor-related Apoptosis-inducing Ligand Receptor 3; CFH, Complement Factor H; IL18, interleukin 18; MCP1, Monocyte Chemotactic Protein 1; RANTES, Regulated Upon Activation, Normally T-Expressed, And Presumably Secreted; TNFR-II, Tumor Necrosis Factor Receptor 2; IL1ra, Interleukin 1 Receptor Antagonist; CRP, C-Reactive Protein. *Significant associations between the marker and fasting glucose. Adjusted for age, sex, BMI, waist circumference (WC), Total Cholesterol, HDL, medication for hypertension, smoking, prevalent CVD, lipid lowering medication

**Fig. 2:**
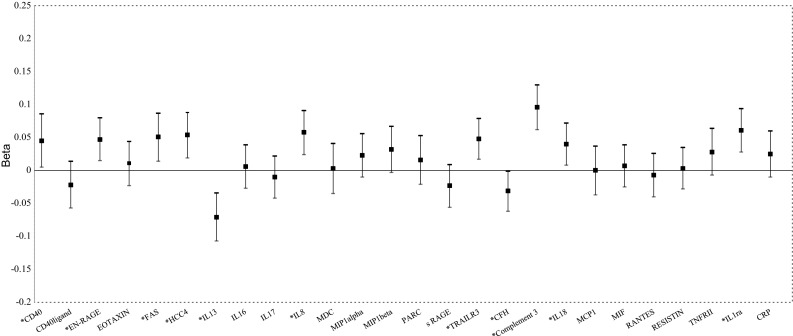
Associations of inflammatory markers with fasting insulin. CD40, cluster of differentiation 40; CD40 ligand, cluster of differentiation 40 ligand; EN-RAGE, Extracellular Newly identified Receptor for Advanced Glycation End-products binding protein; FAS, Fas Cell Surface Death Receptor; HCC4, Human CC chemokine-4; IL13, interleukin 13; IL16, interleukin 16; IL17, interleukin 17; IL8, interleukin 8; MDC, Monocyte Derived Chemokine; MIP1alpha, Macrophage Inflammatory Protein 1 alpha; MIP1beta, Macrophage Inflammatory Protein 1 beta; PARC, Pulmonary and Activation-Regulated Chemokine; sRage, Soluble Receptor of Advanced Glycation End-products; TRAILR3, Tumor Necrosis Factor-related Apoptosis-inducing Ligand Receptor 3; CFH, Complement Factor H; IL18, interleukin 18; MCP1, Monocyte Chemotactic Protein 1; RANTES, Regulated Upon Activation, Normally T-Expressed, And Presumably Secreted; TNFR-II, Tumor Necrosis Factor Receptor 2; IL1ra, Interleukin 1 Receptor Antagonist; CRP, C-Reactive Protein. ^*^Significant associations between the marker and fasting insulin. Adjusted for age, sex, BMI, waist circumference (WC), Total Cholesterol, HDL, medication for hypertension, smoking, prevalent CVD, lipid lowering medication

We used linear regression to investigate the association between each inflammatory marker and fasting glucose and fasting insulin in 851 subjects free of diabetes at baseline (excluding 120 prevalent diabetes cases from 971 subjects with available data) as presented at Figs. 1, 2, Supplementary Table 2.1, Supplementary Table 2.2. Also the associations between markers with HOMA-IR and HOMA-B were investigated using linear regression (Supplementary Table 3). Markers with a right-skewed distribution were transformed to the natural logarithmic scale (including fasting glucose and insulin). For a better comparison between the inflammatory markers, all markers were standardized by dividing the measured value by the standard deviation. We defined marker values as an outlier when the value was >4 standard deviations higher or lower than the mean of the normal variable (not natural log transformed). Participants were excluded from the analyses when the marker value for this person was an outlier. A multiple imputation procedure was used for missing covariates (N = 5 imputations). The analyses with incident pre-diabetes, incident type 2 DM and need for insulin therapy were performed using Cox proportional hazard models to calculate hazard ratios (HRs) and 95% confidence intervals (CI). The first model with incident pre-diabetes and diabetes was adjusted for age and sex (Table 2). Significant markers were further investigated in multivariable models (Table 3). In the second model, we additionally adjusted for body mass index, waist circumference, total cholesterol, HDL-cholesterol, medication for hypertension, smoking, prevalent cardiovascular disease and lipid lowering medication. In the third model we additionally adjusted for C-reaction protein (CRP) levels (except for CRP marker). We sought to investigate the associations between the inflammatory markers and the need for insulin therapy in 115 prevalent diabetes cases with no prevalent use of insulin at baseline (from 120 prevalent cases in total). The inflammatory markers were not correlated to each other, representing 26 independent variables. As a sensitivity analysis, to identify the most robust findings in every analysis, we applied a Bonferroni corrected *p* value of 1.9 × 10^−3^ (0.05/26 markers).The analyses were performed using IBM SPSS Statistics for Windows (IBM SPSS Statistics for Windows, Armonk, New York: IBM Corp) and R V.3.0.1 (R Foundation for Statistical Computing, Vienna, Austria).

## Results

Table 1 summarizes the baseline characteristics of 971 participants, including 120 prevalent diabetes cases. The mean (SD) age at baseline was 73.0 (7.5) years and 44.8% of our population sample were males. The mean BMI (SD) was 26.7 (3.9) kg/m^2^ and 12.6% of the study population used statin.

**Table 1:** Baseline characteristics of study participants

Characteristic	Value^a^
Total population number	971
Age (years)	73.0 ± 7.5
Men, n (%)	435.0 (44.8)
Waist circumference (m)	0.9 ± 0.1
Body mass index (kg/m^2^)	26.7 ± 3.9
Systolic blood pressure (mmHg)	144.0 ± 21.7
Diastolic blood pressure (mmHg)	75.0 ± 11.0
Hypertension medication with indication, n (%)	744.0 (76.6)
Total cholesterol (mmol/L)	5.8 ± 1.0
HDL cholesterol (mmol/L)	1.4 ± 0.4
Fasting glucose (mmol/L)	5.6 (3.54)
Fasting insulin (uIU/L)	9.4 (19.87)
Current smokers, n (%)	137.0 (14.1)
Former smokers, n (%)	483.0 (49.7)
Prevalent CVD, n (%)	201.0 (20.7)
Alcohol intake in drinkers (76%) (g/day)	5.71 (42.73)
Lipid lowering medication, n (%)	122.0 (12.6)

HDL, high density lipoproteins; CVD, cardiovascular disease

^a^Plus-minus values are mean ± standard deviation or median (inter-quartile range)

Baseline levels of inflammation markers are presented in Supplementary Table 1.4.

### Cross-sectional analysis

Figures 1 and 2 present the multivariable adjusted associations between the inflammatory markers and fasting glucose, fasting insulin in 851 subjects free of diabetes at baseline. Three markers, EN-RAGE, IL13 and sRAGE were significantly associated with fasting glucose. CD40, EN-RAGE, FAS, HCC4, IL13, IL18, TRAILR3, CFH, complement 3, IL18 and IL1ra were significantly associated with fasting insulin.

### Prospective analyses

During a median follow-up of 9.5 years in 698 subjects free of pre-diabetes at baseline, 139 cases of pre-diabetes were identified (21 pre-diabetes cases per 1000 person-years). Supplementary Table 1.1 presents baseline characteristics among pre-diabetes cases and non-cases.

In age and sex adjusted model, EN-RAGE, IL13, CFH, IL18 and CRP were associated with incident pre-diabetes (Table 2). In multivariate models, IL13 (HR = 0.77), EN-RAGE (HR = 1.23) and CRP (HR = 1.26) remained associated with incident pre-diabetes (Table 3).

**Table 2:** Age and sex-adjusted associations between markers and incident pre-diabetes, incident type 2 diabetes mellitus

Marker	Incident pre-diabetes		Incident diabetes	
	HR (95% CI)	*P* value	HR (95% CI)	*P* value
CD40 (ng/mL)	0.93 (0.72, 1.19)	0.5	1.18 (0.91, 1.52)	0.2
CD40 ligand^a^ (ng/mL)	0.95 (0.79, 1.16)	0.6	1.06 (0.85, 1.32)	0.6
EN-RAGE^a^ (ng/mL)	1.30 (1.08, 1.56)	**5.0** × **10** ^**−3**^	1.25 (1.01, 1.54)	**4.0** × **10** ^**−2**^
Eotaxin^a^ (pg/mL)	0.95 (0.79, 1.15)	0.6	0.98 (0.80, 1.21)	0.8
FAS^a^ (ng/mL)	1.09 (0.88, 1.35)	0.4	1.09 (0.87, 1.38)	0.4
HCC4 (ng/mL)	1.11 (0.90, 1.35)	0.3	1.24 (0.99, 1.53)	5.4 **×** 10^−2^
IL13^a^ (pg/mL)	0.78 (0.64, 0.94)	**8.0** × **10** ^**−3**^	0.62 (0.50, 0.76)	^**b**^ **5.0** × **10** ^**−6**^
IL16 (pg/mL)	1.07 (0.88, 1.29)	0.4	1.17 (0.94, 1.45)	0.1
IL17^a^ (pg/mL)	0.97 (0.81, 1.16)	0.7	0.75 (0.62, 0.91)	**3.0** × **10** ^**−3**^
IL8^a^ (pg/mL)	1.05 (0.87, 1.27)	0.5	1.19 (0.97, 1.47)	0.1
MDC (pg/mL)	0.94 (0.75, 1.17)	0.5	1.19 (0.94, 1.50)	0.1
MIP1 alpha^a^ (pg/mL)	1.09 (0.90, 1.32)	0.3	1.08 (0.87, 1.34)	0.5
MIP1 beta^a^ (pg/mL)	1.05 (0.87, 1.26)	0.6	1.00 (0.81, 1.25)	0.9
PARC (ng/mL)	1.08 (0.88, 1.32)	0.4	0.94 (0.75, 1.19)	0.6
sRAGE^a^ (ng/mL)	0.95 (0.79, 1.14)	0.6	0.91 (0.75, 1.11)	0.3
TRAILR3^a^ (ng/mL)	1.18 (0.98, 1.41)	8.1 **×** 10^−2^	1.19 (0.97, 1.47)	9.1 **×** 10^−2^
CFH^a^ (ug/mL)	1.24 (1.02, 1.49)	**2.8** **×** **10** ^**−2**^	1.05 (0.87, 1.28)	0.6
Complement 3^a^ (mg/mL)	1.13 (0.94, 1.36)	0.1	1.44 (1.17, 1.77)	^**b**^ **1.0** × **10** ^**−3**^
IL18^a^ (pg/mL)	1.22 (1.02, 1.47)	**3.2** × **10** ^**−2**^	1.35 (1.10, 1.65)	**4.0** × **10** ^**−3**^
MCP1^a^ (pg/mL)	0.93 (0.76, 1.14)	0.4	0.99 (0.79, 1.25)	0.9
MIF^a^ (ng/mL)	0.97 (0.82, 1.14)	0.6	1.11 (0.92, 1.35)	0.2
RANTES^a^ (ng/mL)	0.89 (0.75, 1.05)	0.1	1.05 (0.87, 1.27)	0.6
Resistin^a^ (ng/mL)	1.02 (0.85, 1.24)	0.8	0.96 (0.78, 1.18)	0.7
TNFRII^a^ (ng/mL)	0.97 (0.79, 1.18)	0.7	1.27 (1.03, 1.58)	**2.9** × **10** ^**−2**^
Il1ra^a^ (pg/mL)	1.04 (0.87, 1.25)	0.6	1.24 (1.02, 1.51)	**3.4** × **10** ^**−2**^
CRP^a^ (ug/mL)	1.32 (1.10, 1.58)	**3.0** × **10** ^**−3**^	1.64 (1.33, 2.02)	^**b**^ **4.0** × **10** ^**−6**^

CD40, cluster of differentiation 40; CD40 ligand, cluster of differentiation 40 ligand; EN-RAGE, Extracellular Newly identified Receptor for Advanced Glycation End-products binding protein; FAS, Fas Cell Surface Death Receptor; HCC4, Human CC chemokine-4; IL13, interleukin 13; IL16, interleukin 16; IL17, interleukin 17; IL8, interleukin 8; MDC, Monocyte Derived Chemokine; MIP1alpha, Macrophage Inflammatory Protein 1 alpha; MIP1beta, Macrophage Inflammatory Protein 1 beta; PARC, Pulmonary and Activation-Regulated Chemokine; sRage, Soluble Receptor of Advanced Glycation End-products; TRAILR3, Tumor Necrosis Factor-related Apoptosis-inducing Ligand Receptor 3; CFH, Complement Factor H; IL18, interleukin 18; MCP1, Monocyte Chemotactic Protein 1; RANTES, Regulated Upon Activation, Normally T-Expressed, And Presumably Secreted; TNFR-II, Tumor Necrosis Factor Receptor 2; IL1ra, Interleukin 1 Receptor Antagonist; CRP, C-Reactive Protein

Bold values indicate the significance level of *P* ≤ 0.05

^a^Naturally log-transformed

^b^Sensitivity analysis: significant after Bonferroni correction (*p* = 0.05/26 = 1.9 × 10^−3^)

**Table 3:** Multivariable-adjusted associations between markers and incident pre-diabetes, incident type 2 diabetes mellitus

Marker	Incident pre-diabetes		Incident type 2 diabetes	
	HR (95%CI)	P-value	HR (95%CI)	P-value
IL13				
Model 1	0.78 (0.64, 0.94)	**8.0** **×** **10** ^**−3**^	0.62 (0.50, 0.76)	**5.0** **×** **10** ^**−6**^
Model 2	0.78 (0.63, 0.98)	**2.9** **×** **10** ^**−2**^	0.68 (0.53, 0.88)	**4.0** **×** **10** ^**−3**^
Model 3	0.77 (0.62, 0.96)	**2.2** **×** **10** ^**−2**^	0.67 (0.52, 0.86)	**2.0** **×** **10** ^**−3**^
IL17				
Model 1	0.97 (0.81, 1.16)	0.7	0.75 (0.62, 0.91)	**3.0** **×** **10** ^**−3**^
Model 2	0.97 (0.82, 1.16)	0.7	0.75 (0.62, 0.91)	**4.0** **×** **10** ^**−3**^
Model 3	0.98 (0.82, 1.17)	0.8	0.76 (0.63, 0.93)	**7.0** **×** **10** ^**−3**^
EN-RAGE				
Model 1	1.30 (1.08, 1.56)	**5.0** **×** **10** ^**−3**^	1.25 (1.01, 1.54)	**4.0** **×** **10** ^**−2**^
Model 2	1.28 (1.06, 1.56)	**1.2** **×** **10** ^**−2**^	1.13 (0.89, 1.41)	0.3
Model 3	1.23 (1.01, 1.51)	**4.1** **×** **10** ^**−2**^	1.05 (0.83, 1.32)	0.6
Complement 3				
Model 1	1.13 (0.94, 1.36)	0.1	1.44 (1.17, 1.77)	**1.0** **×** **10** ^**−3**^
Model 2	1.05 (0.86, 1.27)	0.6	1.19 (0.96, 1.49)	0.1
Model 3	0.99 (0.82, 1.21)	0.9	1.10 (0.87, 1.39)	0.4
CFH				
Model 1	1.24 (1.02, 1.49)	**2.8** **×** **10** ^**−2**^	1.05 (0.87, 1.28)	0.6
Model 2	1.19 (0.99, 1.45)	6.5 **×** 10^−2^	0.98 (0.81, 1.18)	0.8
Model 3	1.18 (0.97, 1.42)	9.7 **×** 10^−2^	0.98 (0.81, 1.18)	0.8
IL18				
Model 1	1.22 (1.02, 1.47)	**3.2** **×** **10** ^**−2**^	1.35 (1.10, 1.65)	**4.0** **×** **10** ^**−3**^
Model 2	1.17 (0.97, 1.41)	0.1	1.22 (0.98, 1.50)	6.7 **×** 10^−2^
Model 3	1.13 (0.94, 1.36)	0.1	1.18 (0.96, 1.46)	0.1
TNFRII				
Model 1	0.97 (0.79, 1.18)	0.7	1.27 (1.03, 1.58)	**2.9** **×** **10** ^**−2**^
Model 2	0.89 (0.73, 1.09)	0.2	1.08 (0.86, 1.37)	0.5
Model 3	0.81 (0.66, 1.01)	6.1 **×** 10^−2^	0.99 (0.78, 1.28)	0.9
IL1ra				
Model 1	1.04 (0.87, 1.25)	0.6	1.24 (1.02, 1.51)	**3.4** **×** **10** ^**−2**^
Model 2	0.97 (0.80, 1.17)	0.7	1.03 (0.83, 1.27)	0.8
Model 3	0.94 (0.78, 1.14)	0.5	0.98 (0.79, 1.22)	0.8
CRP				
Model 1	1.32 (1.10, 1.58)	**3.0** **×** **10** ^**−3**^	1.64 (1.33, 2.02)	**4.0** **×** **10** ^**−6**^
Model 2	1.26 (1.04, 1.53)	**1.8** **×** **10** ^**−2**^	1.32 (1.05, 1.67)	**1.7** **×** **10** ^**−2**^
Model 3	NA	NA	NA	NA

Bold values indicate the significance level of *P* ≤ 0.05

(1) Age and sex adjusted

(2) Additionally adjusted for BMI, waist circumference (WC), Total Cholesterol, HDL, medication for hypertension, smoking, prevalent CVD, lipid lowering medication

(3) Additionally adjusted for CRP

During a median follow-up of 12.1 years in 851 subjects free of diabetes at baseline, 110 cases of incident type 2 diabetes were identified (11 diabetes cases per 1000 person-years). Supplementary Table 1.2 presents baseline characteristics among diabetes cases and non-cases.

In age and sex adjusted model, EN-RAGE, IL13, IL17, complement 3, IL18, TNFRII, IL1ra and CRP were associated with incident type 2 diabetes (Table 2).

In multivariate models, IL13 (HR = 0.67), IL17 (HR = 0.76) and CRP (HR = 1.32) remained associated with incident type 2 diabetes (Table 3).

During a median follow-up of 7.5 years in 115 prevalent diabetics free of insulin at baseline, 26 started insulin therapy (30 insulin starters per 1000 person-years). Supplementary Table 1.3 presents baseline characteristics among insulin starters and non-starters.

The only marker associated with need for insulin therapy was IL13. In age and sex adjusted model, the risk for insulin therapy start was 45% lower per standard deviation increase in the natural log-transformed IL13 (HR = 0.55, 95% CI: 0.34, 0.90), (Supplementary Table 4). The association between 1L13 and initiation of insulin therapy remained significant after further adjustment for BMI, waist circumference, total cholesterol, HDL, medication for hypertension, smoking, prevalent CVD, lipid lowering medication (HR = 0.49, 95% CI: 0.28, 0.91).

## Discussion

Although a sizable number of studies have documented the association of inflammatory markers with type 2 DM, most of them investigated the risk to become diabetic, but not the risk of pre-diabetes and insulin therapy start [8]. In this study we investigated a wide range of inflammatory markers for phase-specific prediction of progression to type 2 DM and identified EN-RAGE, IL13 and IL17 as novel inflammatory markers. Higher EN-RAGE levels were associated with an increased risk of incident pre-diabetes, whereas higher IL13 levels were associated with a decreased risk of pre-diabetes, incident type 2 DM and need for insulin therapy. Higher IL17 levels were associated with a decreased risk of incident type 2 DM. In addition, this study reconfirm the previously found associations between high CRP levels and the increased risk for type 2 diabetes [6–8].

EN-RAGE, also known as S100A12 or Calgranulin C, is a calcium-binding pro inflammatory protein mainly secreted by granulocytes. The best known target protein of EN-RAGE are RAGE (Receptor for Advanced Glycation Endproducts) [22] and TLR4 (Toll-like receptor 4) [23]. Ligation of EN-RAGE with RAGE or TLR4, which are both gatekeepers of the innate immune system, activates inflammatory cascades, including the NF-κB pathway and JNK (c-Jun NH_2_–terminal kinase) [24]. NF-κB and JNK are both signaling pathways involved in the pathogenesis of insulin resistance and type 2 DM [25]. EN-RAGE is positively associated with chronic inflammatory disorders such as inflammatory bowel disease, chronic kidney disease, subclinical atherosclerosis and coronary artery disease. A cross-sectional study in Italian population found that prediabetic patients exhibited lower RAGE plasma levels as well as increased levels of proinflammatory S100A12 in both prediabetic and diabetic patients [26]. In addition, we have previously reported the positive association between EN-RAGE and incident CHD in the Rotterdam Study [19]. Kosaki et al. [27] observed increased plasma EN-RAGE levels in patients with type 2 DM. EN-RAGE was significantly associated with both HOMA-IR and HOMA-B, suggesting proinflammatory EN-RAGE leads to incident type 2 DM via inflammation-induced insulin resistance as well as via B-cell dysfunction (Supplementary Table 3).

Interleukin 13 (IL13) is a cytokine mainly produced by the T-helper (Th)-2 subset of lymphocytes, but also from non-T-cell populations such as mast cells, basophils, dendritic cells, keratinocytes and eosinophils [28–30]. IL13 is a regulator of inflammation and immune responses [31]. IL13 has a common receptor unit (α-chain) with interleukin 4 (IL4), which explains the similarities between IL13 and IL4 [32]. Previous research has reported a preventive effect of IL4 on the onset of diabetes in non-obese diabetic mice (NOD mice) [33]. Furthermore, Zaccone et al. [34] found that IL13 prevents autoimmune diabetes in NOD mice, providing evidence that IL13 down-regulates the immune-inflammatory diabetogenic pathways, which is in agreement with our findings. Wong et al. [35] suggested the stimulation of IL13 receptors on T-cells, as a new pathway for tolerance induction in NOD mice. In addition, IL13 is a B cell stimulating factor, which further supports our observation [36]. Stanya et al. [37] conclude that IL13 mitigates proinflammatory response in mice and regulates glucose homeostasis via the IL-13rα1–STAT3 signaling pathway in the liver, and that this pathway might provide a target for glycemic control in type 2 DM.

We also investigated the associations of IL13 with HOMA-IR and HOMA-B. IL13 was associated with both of them, suggesting a protective role against insulin resistance and B-cell dysfunction (Supplementary Table 3).

There are six members in the interleukin 17 (IL17) cytokine family, including IL17A, IL17B, IL17C, IL17D, IL17E (also known as IL25) and IL17F. Among all the members, the biological function and regulation of IL17A and IL17F are best understood. IL17A was produced mainly in T cells, whereas IL-17F was produced in T cells, innate immune cells, and epithelial cells. Functionally, both IL17A and IL17F mediate pro-inflammatory responses [38, 39]. IL17 family cytokines have been linked to many autoimmune diseases, including multiple sclerosis, rheumatoid arthritis, inflammatory bowel disease and psoriasis [40]. The role of IL17 on the risk for type 2 DM remains unclear. Roohi A et al. [41] reported no association between serum IL17 levels and type 1 and 2 diabetes. Another study found that therapeutic improvement of glucoregulation in newly diagnosed type 2 DM patients is associated with a reduction of IL-17 levels [42]. Our study suggest a protective IL17 cytokine against the risk for type 2 DM (HR = 0.76), which is controversial and novel to the already known pro-inflammatory role of IL17 family cytokines. However, a cross-sectional case–control study has reported inverse associations of serum levels of IL17 with type 2 DM as well as with retinopathy, which is in line with our findings [43].

This study has certain strengths and limitations. To our knowledge, this is the first prospective population-based cohort study to investigate the association between a large set of novel inflammatory markers and the progression of type 2 DM with long-term follow-up. Furthermore, we performed sensitivity analysis, adjusting the type I error for multiple testing in order to highlight the most robust associations in every analyses. However, given the novelty of the markers, we reported significant findings at the level of 0.05 to avoid missing possibly important findings [44]. Beyond the identification of new novel inflammatory markers for type 2 diabetes, our findings relate them specifically to different stages of the disease. We are also aware of some limitations of the study. First, we had to exclude inflammatory markers with very low serum concentrations. However, the selected markers have >60% completeness of measurements, indicating acceptable quality of quantification. Second, our population is 55 years and older, thus generalization of the results to a younger age should be done with caution. Also, the Rotterdam Study mainly includes individuals from European Ancestry (98%). The effect estimates might differ between ethnicities.

A better prevention of type 2 DM requires the targeting of subjects at high risk in very early phases, such as pre-diabetes [12]. In this context, it is worth to investigate novel inflammatory markers that might be detectors of different stages of type 2 DM development [17].

In conclusion, our results show various inflammatory markers are associated with the progression from normoglycemia to type 2 DM and need for insulin therapy in a phase-specific manner. Among them, EN-RAGE is a novel inflammatory marker for pre-diabetes, IL17 for incident type 2 DM and IL13 for pre-diabetes, incident type 2 DM and insulin therapy start. This study only indicates new associations, emphasizing the need for further studies to establish the role of EN-RAGE, IL13 and IL17 in the development of type 2 diabetes.

## Electronic supplementary material

Below is the link to the electronic supplementary material.

Supplementary material 1 (DOCX 56 kb)
