# Supplementary material for: Novel inflammatory markers for incident pre-diabetes and type 2 diabetes: the Rotterdam Study
Source: Eur J Epidemiol. 2017 Mar 3;32(3):217–26. doi: 10.1007/s10654-017-0236-0 (PMC5380703; doi:10.1007/s10654-017-0236-0)
Supplement: Supplementary file 1 — Supplementary material 1 (DOCX 56 kb) [file 10654_2017_236_MOESM1_ESM.docx]

**Online Supplementary data**

**Supplementary table 1.1** **Baseline characteristics among non- pre-diabetes cases/ pre-diabetes cases.**

| **Characteristic** | **Non- pre-diabetes cases** | **Incident pre-diabetes cases** | **p-value** |
| --- | --- | --- | --- |
| Total population number | 559 | 139 |  |
| Age, years | 73 ± 7.6 | 71 ± 6.1 | **< 0.001** |
| Men, n (%) | 238 (43) | 60 (43.2) | 0.9 |
| Waist Circumference, m | 0.9 ± 0.1 | 0.9 ± 0.1 | 0.3 |
| Body mass index, kg/m^2^ | 25.9 ± 3.6 | 27.0 ± 3.8 | **0.003** |
| Systolic blood pressure, mmHg | 143 ± 21.7 | 142 ± 20.1 | 0.4 |
| Diastolic blood pressure, mmHg | 74.6 ± 10.9 | 74.9 ± 10.9 | 0.7 |
| Hypertension medication with indication, n(%) | 107 (19.3) | 30 (21.6) | **0.052** |
| Total cholesterol, mmol/L | 5.8 ± 1.0 | 5.8 ± 0.9 | 0.9 |
| HDL cholesterol, mmol/L | 1.5 ± 0.4 | 1.4 ± 0.4 | **0.016** |
| Fasting glucose, mmol/L | 5.3 (4.7 – 5.9) | 5.6 (4.9 – 6.0) | **< 0.001** |
| Fasting insulin, uIU/L | 8.2 (3.7 – 18.4) | 9.6 (3.7 – 22.4) | **0.002** |
| Current smokers, n (%) | 85 (15.4) | 19 (13.7) | 0.6 |
| Former smokers, n (%) | 255 (46.1) | 77 (55.4) | 0.1 |
| Prevalent CVD, n (%) | 94 (17) | 15 (10.8) | 0.07 |
| Alcohol intake in drinkers (76%), g/day | 2.9 (0.0 – 40.2) | 2.9 (0.0 – 40.0) | 0.6 |
| Lipid lowering medication, n (%) | 57 (10.3) | 26 (18.7) | **0.007** |

Abbreviations: HDL, high density lipoproteins; CVD, cardiovascular disease.

Plus-minus values are means ± standard deviation or median (inter-quartile range).

**Supplementary table 1**.**2** **Baseline characteristics among non- diabetes cases/ diabetes cases.**

| **Characteristic** | **Non- diabetes cases** | **Incident diabetes cases** | **p-value** |
| --- | --- | --- | --- |
| Total population number | 741 | 110 |  |
| Age, years | 73 ± 7.5 | 70 ± 5.9 | **< 0.001** |
| Men, n (%) | 330 (44.9) | 46 (41.8) | 0.5 |
| Waist Circumference, m | 0.9 ± 0.1 | 0.9 ± 0.1 | **< 0.001** |
| Body mass index, kg/m^2^ | 26.2 ± 3.7 | 28.4 ± 4.2 | **< 0.001** |
| Systolic blood pressure, mmHg | 143.4 ± 21.7 | 144.9 ± 19.6 | 0.4 |
| Diastolic blood pressure, mmHg | 75.0 ± 11.2 | 75.7 ± 11.2 | 0.5 |
| Hypertension medication with indication, n(%) | 154 (21) | 36 (32.7) | **0.02** |
| Total cholesterol, mmol/L | 5.8 ± 1.0 | 5.9 ± 0.9 | 0.4 |
| HDL cholesterol, mmol/L | 1.4 ± 0.4 | 1.3 ± 0.4 | **< 0.001** |
| Fasting glucose, mmol/L | 5.4 (4.7 – 6.3) | 6.1 (5.1 – 6.8) | **< 0.001** |
| Fasting insulin, uIU/L | 8.5 (3.7 – 19.3) | 12.9 (5.3 – 27.3) | **< 0.001** |
| Current smokers, n (%) | 107 (14.6) | 18 (16.4) | 0.6 |
| Former smokers, n (%) | 355 (48.3) | 57 (51.8) | 0.6 |
| Prevalent CVD, n (%) | 124 (16.9) | 9 (8.2) | **0.02** |
| Alcohol intake in drinkers (76%), g/day | 2.9 (0.0 – 41.9) | 2.9 (0.0 – 40.1) | 0.5 |
| Lipid lowering medication, n (%) | 81 (11) | 14 (12.7) | 0.8 |

Abbreviations: HDL, high density lipoproteins; CVD, cardiovascular disease.

Plus-minus values are means ± standard deviation or median (inter-quartile range).

**Supplementary table 1**.**3** **Baseline characteristics among non-insulin starters/ insulin starters.**

| **Characteristic** | **Non-insulin starters** | **Insulin starters** | **p-value** |
| --- | --- | --- | --- |
| Total population number | 89 | 26 |  |
| Age, years | 74.9 ± 8.3 | 73.6 ± 6.5 | **0.001** |
| Men, n (%) | 45 (50.6) | 12 (46.2) | 0.3 |
| Waist Circumference, m | 0.9 ± 0.1 | 0.9 ± 0.1 | 0.6 |
| Body mass index, kg/m^2^ | 28.3 ± 4.4 | 28.7 ± 4.7 | 0.09 |
| Systolic blood pressure, mmHg | 145.8 ± 21.0 | 149.7 ± 28.5 | 0.7 |
| Diastolic blood pressure, mmHg | 75.3 ± 10.1 | 75.4 ± 9.5 | 0.7 |
| Hypertension medication with indication, n(%) | 39 (43.8) | 8 (30.8) | 0.5 |
| Total cholesterol, mmol/L | 5.7 ± 0.9 | 5.8 ± 0.8 | 0.5 |
| HDL cholesterol, mmol/L | 1.2 ± 0.4 | 1.1 ± 0.2 | 0.2 |
| Fasting glucose, mmol/L | 7.5 (5.4 – 12.5) | 8.8 (6.6 – 17.2) | **0.02** |
| Fasting insulin, uIU/L | 12.2 (4.6 – 41.1) | 12.7 (5.9 – 52.9) | 0.7 |
| Current smokers, n (%) | 11 (12.4) | 0 (0) | **0.03** |
| Former smokers, n (%) | 49 (55.1) | 17 (65.4) | 0.1 |
| Prevalent CVD, n (%) | 22 (24.7) | 8 (30.8) | **0.005** |
| Alcohol intake in drinkers (76%), g/day | 1.4 (0.0 – 21.1) | 1.4 (0.0 – 18.1) | 0.4 |
| Lipid lowering medication, n (%) | 15 (16.9) | 8 (30.8) | 0.6 |

Abbreviations: HDL, high density lipoproteins; CVD, cardiovascular disease.

Plus-minus values are means ± standard deviation or median (inter-quartile range).

**Supplementary table 1**.**4** **Baseline characteristics of the inflammatory markers.**

| **Marker** | **Value** |
| --- | --- |
| Total population free of diabetes | 851 |
| CD40, ng/mL | 0.73 ± 0.27 |
| CD40 ligand ^*^, ng/mL | 0.03 (0.01 – 0.06) |
| EN-RAGE ^*^, ng/mL | 10.70 (4.82 – 24.45) |
| Eotaxin ^*^, pg/mL | 161.00 (65.40 – 330.65) |
| FAS ^*^, ng/mL | 4.65 (2.94 – 8.18) |
| HCC4, ng/mL | 4.87 ± 1.95 |
| IL13 ^*^, pg/mL | 76.20 (48.70 - -123.00) |
| IL16, pg/mL | 381.98 ± 103.89 |
| IL17 ^*^, pg/mL | 12.90 (6.22 – 23.30) |
| IL8 ^*^, pg/mL | 9.24 (4.25 – 20.92) |
| MDC, pg/mL | 365.61 ± 124.01 |
| MIP1 alpha ^*^, pg/mL | 46.20 (27.22 – 73.46) |
| MIP1 beta ^*^, pg/mL | 122.00 (66.06 – 323.20) |
| PARC, ng/mL | 29.93 ± 11.12 |
| sRAGE ^*^, ng/mL | 2.67 (1.27 – 5.66) |
| TRAILR3 ^*^, ng/mL | 6.55 (3.48 – 12.48) |
| CFH ^*^, ug/mL | 2520 (890.95 – 3700) |
| Complement 3^*^, mg/mL | 0.82 (0.62 – 1.06) |
| IL18^*^, pg/mL | 187 (100 – 381.40) |
| MCP1^*^, pg/mL | 184 (113 – 309) |
| MIF^*^, ng/mL | 0.06 (0.01 – 0.15) |
| RANTES^*^, ng/mL | 0.51 (0.18 – 1.79) |
| Resistin^*^, ng/mL | 0.42 (0.17 – 0.99) |
| TNFRII^*^, ng/mL | 3.51 (2.25 – 6.21) |
| Il1ra^*^, pg/mL | 66.80 (25.99 – 191) |
| CRP^*^, ug/mL | 1.37 (0.23 – 8.90) |

Plus-minus values are means ± standard deviation or median (inter-quartile range). **^*^** Naturally log-transformed markers. CD40, cluster of differentiation 40; CD40 ligand, cluster of differentiation 40 ligand ; EN-RAGE, Extracellular Newly identified Receptor for Advanced Glycation End-products binding protein; FAS, Fas Cell Surface Death Receptor; HCC4, Human CC chemokine-4; IL13, interleukin 13; IL16, interleukin 16; IL17, interleukin 17; IL8, interleukin 8; MDC, Monocyte Derived Chemokine; MIP1alpha, Macrophage Inflammatory Protein 1 alpha; MIP1beta, Macrophage Inflammatory Protein 1 beta; PARC, Pulmonary and Activation-Regulated Chemokine; sRage, Soluble Receptor of Advanced Glycation End-products; TRAILR3, Tumor Necrosis Factor-related Apoptosis-inducing Ligand Receptor 3; CFH, Complement Factor H; IL18, interleukin 18; MCP1, Monocyte Chemotactic Protein 1; RANTES, Regulated Upon Activation, Normally T-Expressed, And Presumably Secreted; TNFR-II, Tumor Necrosis Factor Receptor 2; IL1ra, Interleukin 1 Receptor Antagonist; CRP, C-Reactive Protein.

**Supplementary table 2.1 Age and sex adjusted associations between markers of inflammation and fasting glucose and insulin.**

| **Marker** | **N** | **Fasting glucose** | | **Fasting insulin** | |
| --- | --- | --- | --- | --- | --- |
|  |  | **Beta (95%CI)** | **P-value** | **Beta (95%CI)** | **P-value** |
| CD40, ng/mL | 848 | 0.009 (0.001, 0.018) | **3.2 × 10^-2^** | 0.097 (0.050, 0.143) | **^b^4.4 × 10^-5^** |
| CD40 ligand ^*^, ng/mL | 780 | -0.001 (-0.008, 0.007) | 0.8 | -0.018 (-0.060, 0.024) | 0.3 |
| EN-RAGE ^*^, ng/mL | 843 | 0.012 (0.005, 0.019) | **^b^1 × 10^-3^** | 0.078 (0.041, 0.12) | **^b^3.6 x 10^-5^** |
| Eotaxin ^*^, pg/mL | 841 | -0.005 (-0.012, 0.002) | 0.1 | -0.032 (-0.070, 0.007) | 0.1 |
| FAS ^*^, ng/mL | 837 | 0.004 (-0.003, 0.012) | 0.2 | 0.113 (0.072, 0.153) | **^b^7.0 × 10^-8^** |
| HCC4, ng/mL | 850 | 0.008 (0.001, 0.016) | **2.6 × 10^-2^** | 0.098 (0.058, 0.138) | **^b^1.0 × 10^-6^** |
| IL13 ^*^, pg/mL | 814 | -0.014 (-0.021, -0.007) | **^b^7.7 × 10^-5^** | -0.155 (-0.190, -0.119) | **^b^9.6 × 10^-17^** |
| IL16, pg/mL | 849 | 0.001 (-0.006, 0.008) | 0.7 | 0.052 (0.015, 0.090) | **7.0 × 10^-3^** |
| IL17 ^*^, pg/mL | 805 | -1 x 10^-4^  (-0.007, 0.007) | 0.9 | -0.026 (-0.063, 0.011) | 0.1 |
| IL8 ^*^, pg/mL | 824 | 0.003 (-0.004, 0.011) | 0.3 | 0.057 (0.019, 0.096) | **4.0 × 10^-3^** |
| MDC, pg/mL | 846 | 2 x 10^-4^  (-0.008, 0.008) | 0.9 | 0.049 (0.006, 0.091) | **2.7 × 10^-2^** |
| MIP1 alpha ^*^, pg/mL | 846 | -1.1 x 10^-4^ (-0.007, 0.007) | 0.9 | 0.052 (0.014, 0.090) | **8.0 × 10^-3^** |
| MIP1 beta ^*^, pg/mL | 844 | 0.001 (-0.007, 0.008) | 0.8 | 0.063 (0.023, 0.103) | **2.0 × 10^-3^** |
| PARC, ng/mL | 845 | 0.002 (-0.005, 0.010) | 0.5 | 0.039 (-0.003, 0.082) | 7.0 **×** 10^-2^ |
| sRAGE ^*^, ng/mL | 847 | -0.012 (-0.019, -0.005) | **^b^1 × 10^-3^** | -0.045 (-0.082, -0.008) | **1.8 × 10^-2^** |
| TRAILR3 ^*^, ng/mL | 844 | 0.003 (-0.003, 0.010) | 0.3 | 0.082 (0.046, 0.118) | **^b^7.0 × 10^-6^** |
| CFH ^*^, ug/mL | 840 | 0.004 (-0.002, 0.011) | 0.1 | -0.001 (-0.037, 0.034) | 0.9 |
| Complement 3^*^, mg/mL | 851 | 0.014 (0.008, 0.021) | **^b^2.3 × 10^-5^** | 0.179 (0.144, 0.213) | **^b^6.0 × 10^-23^** |
| IL18^*^, pg/mL | 844 | 0.003 (-0.004, 0.010) | 0.3 | 0.083 (0.046, 0.119) | **^b^9.0 × 10^-6^** |
| MCP1^*^, pg/mL | 846 | -0.008 (-0.016, -8 x 10^-0.005)^ | **4.7 × 10^-2^** | -0.015 (-0.058, 0.027) | 0.4 |
| MIF^*^, ng/mL | 831 | 4 x 10^-4^ (-0.006, 0.007) | 0.8 | 0.040 (0.004, 0.076) | **3.1 × 10^-2^** |
| RANTES^*^, ng/mL | 849 | -0.003 (-0.010, 0.004) | 0.3 | -0.023 (-0.059, 0.013) | 0.2 |
| Resistin^*^, ng/mL | 844 | -0.005 (-0.012, 0.002) | 0.1 | 0.022 (-0.015, 0.058) | 0.2 |
| TNFRII^*^, ng/mL | 846 | 2 x 10^-4^ (-0.007, 0.007) | 0.9 | 0.105 (0.066, 0.144) | **^b^1.0 × 10^-7^** |
| Il1ra^*^, pg/mL | 821 | 0.011 (0.004, 0.018) | **^b^1 × 10^-3^** | 0.139 (0.104, 0.175) | **^b^1.9 × 10^-14^** |
| CRP^*^, ug/mL | 837 | 0.012 (0.005, 0.019) | **^b^4.8 × 10^-4^** | 0.119 (0.083, 0.156) | **^b^1.1 × 10^-10^** |

^*^ Naturally log-transformed

CD40, cluster of differentiation 40; CD40 ligand, cluster of differentiation 40 ligand ; EN-RAGE, Extracellular Newly identified Receptor for Advanced Glycation End-products binding protein; FAS, Fas Cell Surface Death Receptor; HCC4, Human CC chemokine-4; IL13, interleukin 13; IL16, interleukin 16; IL17, interleukin 17; IL8, interleukin 8; MDC, Monocyte Derived Chemokine; MIP1alpha, Macrophage Inflammatory Protein 1 alpha; MIP1beta, Macrophage Inflammatory Protein 1 beta; PARC, Pulmonary and Activation-Regulated Chemokine; sRage, Soluble Receptor of Advanced Glycation End-products; TRAILR3, Tumor Necrosis Factor-related Apoptosis-inducing Ligand Receptor 3; CFH, Complement Factor H; IL18, interleukin 18; MCP1, Monocyte Chemotactic Protein 1; RANTES, Regulated Upon Activation, Normally T-Expressed, And Presumably Secreted; TNFR-II, Tumor Necrosis Factor Receptor 2; IL1ra, Interleukin 1 Receptor Antagonist; CRP, C-Reactive Protein. ^b^Sensitivity analysis: significant after Bonferroni correction (p = 0.05/26 = 1.9 × 10^-3^)

**Supplementary table 2.2 Multivariable adjusted associations between markers of inflammation and**

**fasting glucose and insulin.**

| **Marker** | **N** | **Fasting glucose** | | **Fasting insulin** | |
| --- | --- | --- | --- | --- | --- |
|  |  | **Beta (95%CI)** | **P-value** | **Beta (95%CI)** | **P-value** |
| CD40, ng/mL | 848 | 0.006 (-0.003, 0.014) | 0.1 | 0.045 (0.005, 0.086) | **2.9 × 10^-2^** |
| CD40 ligand ^*^, ng/mL | 780 | -4 x 10^-4^ (-0.008, 0.007) | 0.9 | -0.022 (-0.057, 0.014) | 0.2 |
| EN-RAGE ^*^, ng/mL | 843 | 0.009 (0.002, 0.015) | **1.2 × 10^-2^** | 0.047 (0.015, 0.080) | **5 × 10^-3^** |
| Eotaxin ^*^, pg/mL | 841 | -0.001 (-0.008, 0.006) | 0.6 | 0.011 (-0.023, 0.044) | 0.5 |
| FAS ^*^, ng/mL | 837 | -0.001 (-0.008, 0.007) | 0.8 | 0.051 (0.014, 0.087) | **6 × 10^-3^** |
| HCC4, ng/mL | 850 | 0.004 (-0.003, 0.012) | 0.2 | 0.054 (0.019, 0.088) | **3 × 10^-3^** |
| IL13 ^*^, pg/mL | 814 | -0.008 (-0.016, 0.000) | **4.7 × 10^-2^** | -0.071 (-0.107, -0.034) | **^b^1.6 × 10^-4^** |
| IL16, pg/mL | 849 | -0.003 (-0.010, 0.004) | 0.3 | 0.006 (-0.027, 0.039) | 0.7 |
| IL17 ^*^, pg/mL | 805 | 0.001 (-0.005, 0.008) | 0.7 | -0.010 (-0.042, 0.022) | 0.5 |
| IL8 ^*^, pg/mL | 824 | 0.004 (-0.003, 0.011) | 0.2 | 0.058 (0.024, 0.091) | **^b^1.0 × 10^-3^** |
| MDC, pg/mL | 846 | -0.004 (-0.012, 0.004) | 0.3 | 0.003 (-0.035, 0.041) | 0.8 |
| MIP1 alpha ^*^, pg/mL | 846 | -0.002 (-0.009, 0.005) | 0.5 | 0.023 (-0.010, 0.056) | 0.1 |
| MIP1 beta ^*^, pg/mL | 844 | -0.002 (-0.009, 0.005) | 0.5 | 0.032 (-0.003, 0.067) | 6.9 **×** 10^-2^ |
| PARC, ng/mL | 845 | -1 x 10^-4^ (-0.008, 0.008) | 0.9 | 0.016 (-0.021, 0.053) | 0.3 |
| sRAGE ^*^, ng/mL | 847 | -0.009 (-0.015, -0.002) | **1.4 × 10^-2^** | -0.023 (-0.056, 0.009) | 0.1 |
| TRAILR3 ^*^, ng/mL | 844 | 4 x 10^-4^ (-0.006, 0.007) | 0.8 | 0.048 (0.017, 0.079) | **3.0 × 10^-3^** |
| CFH ^*^, ug/mL | 840 | 0.001 (-0.005, 0.008) | 0.6 | -0.031 (-0.062, -0.001) | **4.6 × 10^-2^** |
| Complement 3^*^, mg/mL | 851 | 0.005 (-0.002, 0.012) | 0.1 | 0.096 (0.062, 0.130) | **^b^2.3 × 10^-8^** |
| IL18^*^, pg/mL | 844 | -1 x 10^-4^ (-0.007, 0.007) | 0.9 | 0.040 (0.008, 0.072) | **1.5 × 10^-2^** |
| MCP1^*^, pg/mL | 846 | -0.007 (-0.014, 0.001) | 7.5 **×** 10^-2^ | 3 x 10^-4^ (-0.037, 0.037) | 0.9 |
| MIF^*^, ng/mL | 831 | -0.002 (-0.008, 0.005) | 0.5 | 0.007 (-0.025, 0.039) | 0.6 |
| RANTES^*^, ng/mL | 849 | -0.002 (-0.008, 0.005) | 0.6 | -0.007 (-0.040, 0.026) | 0.6 |
| Resistin^*^, ng/mL | 844 | -0.006 (-0.013, 1 x 10^-4^) | 5.6 **×** 10^-2^ | 0.003 (-0.028, 0.035) | 0.8 |
| TNFRII^*^, ng/mL | 846 | -0.005 (-0.013, 0.002) | 0.1 | 0.028 (-0.007, 0.064) | 0.1 |
| Il1ra^*^, pg/mL | 821 | 0.005 (-0.002, 0.012) | 0.2 | 0.061 (0.028, 0.094) | **^b^2.7 × 10^-4^** |
| CRP^*^, ug/mL | 837 | 0.002 (-0.005, 0.010) | 0.4 | 0.025 (-0.010, 0.060) | 0.1 |

^*^ Naturally log-transformed

CD40, cluster of differentiation 40; CD40 ligand, cluster of differentiation 40 ligand ; EN-RAGE, Extracellular Newly identified Receptor for Advanced Glycation End-products binding protein; FAS, Fas Cell Surface Death Receptor; HCC4, Human CC chemokine-4; IL13, interleukin 13; IL16, interleukin 16; IL17, interleukin 17; IL8, interleukin 8; MDC, Monocyte Derived Chemokine; MIP1alpha, Macrophage Inflammatory Protein 1 alpha; MIP1beta, Macrophage Inflammatory Protein 1 beta; PARC, Pulmonary and Activation-Regulated Chemokine; sRage, Soluble Receptor of Advanced Glycation End-products; TRAILR3, Tumor Necrosis Factor-related Apoptosis-inducing Ligand Receptor 3; CFH, Complement Factor H; IL18, interleukin 18; MCP1, Monocyte Chemotactic Protein 1; RANTES, Regulated Upon Activation, Normally T-Expressed, And Presumably Secreted; TNFR-II, Tumor Necrosis Factor Receptor 2; IL1ra, Interleukin 1 Receptor Antagonist; CRP, C-Reactive Protein. Adjusted for age, sex, BMI, waist circumference (WC), Total Cholesterol, HDL, medication for hypertension, smoking, prevalent CVD, lipid lowering medication. ^b^Sensitivity analysis: significant after Bonferroni correction (p = 0.05/26 = 1.9 × 10^-3^).

**Supplementary Table 3 Associations between markers of inflammation and HOMA indices.**

| **Marker** | **N** | **HOMA-IR** | | **HOMA-B** | |
| --- | --- | --- | --- | --- | --- |
|  |  | **Beta (95%CI)** | **P-value** | **Beta (95%CI)** | **P-value** |
| CD40, ng/mL | 848 | 0.105 (0.056, 0.155) | **^b^3.4 × 10^-5^** | 0.077 (0.033, 0.122) | **^b^1.0 × 10^-3^** |
| CD40 ligand ^*^, ng/mL | 780 | -0.020 (-0.065, 0.025) | 0.3 | -0.012 (-0.052, 0.029) | 0.5 |
| EN-RAGE ^*^, ng/mL | 843 | 0.090 (0.050, 0.130) | **^b^1.0 × 10^-5^** | 0.045 (0.009, 0.081) | **1.4 × 10^-2^** |
| Eotaxin ^*^, pg/mL | 841 | -0.036 (-0.077, 0.005) | 8.8 **×** 10^-2^ | -0.019 (-0.056, 0.018) | 0.3 |
| FAS ^*^, ng/mL | 837 | 0.117 (0.073, 0.162) | **^b^1.7 × 10^-7^** | 0.101 (0.062, 0.141) | **^b^5.5 × 10^-7^** |
| HCC4, ng/mL | 850 | 0.107 (0.064, 0.150) | **^b^9.5 × 10^-7^** | 0.076 (0.037, 0.114) | **^b^1.1 × 10^-4^** |
| IL13 ^*^, pg/mL | 814 | -0.168 (-0.206, -0.130) | **^b^5.0 × 10^-17^** | -0.120 (-0.155, -0.085) | **^b^2.0 × 10^-11^** |
| IL16, pg/mL | 849 | 0.054 (0.013, 0.095) | **1.0 × 10^-2^** | 0.051 (0.014, 0.087) | **7.0 × 10^-3^** |
| IL17 ^*^, pg/mL | 805 | -0.027 (-0.067, 0.013) | 0.1 | -0.024 (-0.060, 0.011) | 0.1 |
| IL8 ^*^, pg/mL | 824 | 0.060 (0.019, 0.102) | **4.0 × 10^-3^** | 0.051 (0.014, 0.088) | **7.0 × 10^-3^** |
| MDC, pg/mL | 846 | 0.049 (0.003, 0.095) | **3.8 × 10^-2^** | 0.048 (0.006, 0.089) | **2.4 × 10^-2^** |
| MIP1 alpha ^*^, pg/mL | 846 | 0.051 (0.010, 0.092) | **1.4 × 10^-2^** | 0.054 (0.018, 0.091) | **4.0 × 10^-3^** |
| MIP1 beta ^*^, pg/mL | 844 | 0.063 (0.020, 0.106) | **4.0 × 10^-3^** | 0.063 (0.025, 0.102) | **^b^1.0 × 10^-3^** |
| PARC, ng/mL | 845 | 0.040 (-0.005, 0.086) | 8.4 **×** 10^-2^ | 0.039 (-0.002, 0.080) | 5.9 **×** 10^-2^ |
| sRAGE ^*^, ng/mL | 847 | -0.057 (-0.097, -0.017) | **6.0 × 10^-3^** | -0.009 (-0.045, 0.027) | 0.6 |
| TRAILR3 ^*^, ng/mL | 844 | 0.085 (0.047, 0.124) | **^b^1.5 × 10^-5^** | 0.073 (0.039, 0.108) | **^b^3.0 × 10^-5^** |
| CFH ^*^, ug/mL | 840 | 0.003 (-0.036, 0.041) | 0.8 | -0.013 (-0.047, 0.022) | 0.4 |
| Complement 3^*^, mg/mL | 851 | 0.193 (0.156, 0.230) | **^b^6.3 × 10^-23^** | 0.140 (0.106, 0.174) | **^b^8.8 × 10^-16^** |
| IL18^*^, pg/mL | 844 | 0.085 (0.046, 0.125) | **^b^2.2 × 10^-5^** | 0.077 (0.042, 0.112) | **^b^1.8 × 10^-5^** |
| MCP1^*^, pg/mL | 846 | -0.023 (-0.069, 0.023) | 0.3 | 0.007 (-0.034, 0.048) | 0.7 |
| MIF^*^, ng/mL | 831 | 0.040 (0.001, 0.079) | **4.2 × 10^-2^** | 0.040 (0.005, 0.075) | **2.6 × 10^-2^** |
| RANTES^*^, ng/mL | 849 | -0.026 (-0.065, 0.013) | 0.1 | -0.012 (-0.047, 0.022) | 0.4 |
| Resistin^*^, ng/mL | 844 | 0.017 (-0.023, 0.057) | 0.4 | 0.037 (0.002, 0.073) | **3.9 × 10^-2^** |
| TNFRII^*^, ng/mL | 846 | 0.106 (0.064, 0.148) | **^b^6.9 × 10^-7^** | 0.103 (0.066, 0.141) | **^b^5.0 × 10^-8^** |
| Il1ra^*^, pg/mL | 821 | 0.150 (0.112, 0.189) | **^b^1.9 × 10^-14^** | 0.110 (0.076, 0.145) | **^b^4.4 × 10^-10^** |
| CRP^*^, ug/mL | 837 | 0.132 (0.093, 0.170) | **^b^3.6 × 10^-11^** | 0.086 (0.051, 0.122) | **^b^2.0 × 10^-6^** |

Age and sex adjusted. The number of subjects differs for each marker, after outliers exclusion. ^*^ Naturally log-transformed. CD40, cluster of differentiation 40; CD40 ligand, cluster of differentiation 40 ligand ; EN-RAGE, Extracellular Newly identified Receptor for Advanced Glycation End-products binding protein; FAS, Fas Cell Surface Death Receptor; HCC4, Human CC chemokine-4; IL13, interleukin 13; IL16, interleukin 16; IL17, interleukin 17; IL8, interleukin 8; MDC, Monocyte Derived Chemokine; MIP1alpha, Macrophage Inflammatory Protein 1 alpha; MIP1beta, Macrophage Inflammatory Protein 1 beta; PARC, Pulmonary and Activation-Regulated Chemokine; sRage, Soluble Receptor of Advanced Glycation End-products; TRAILR3, Tumor Necrosis Factor-related Apoptosis-inducing Ligand Receptor 3; CFH, Complement Factor H; IL18, interleukin 18; MCP1, Monocyte Chemotactic Protein 1; RANTES, Regulated Upon Activation, Normally T-Expressed, And Presumably Secreted; TNFR-II, Tumor Necrosis Factor Receptor 2; IL1ra, Interleukin 1 Receptor Antagonist; CRP, C-Reactive Protein. ^b^Sensitivity analysis: significant after Bonferroni correction (p = 0.05/26 = 1.9 × 10^-3^)

**Supplementary table 4 Associations between markers of inflammation and insulin therapy start in diabetics.**

| **Marker** | **N** | **Insulin therapy start** | |
| --- | --- | --- | --- |
|  |  | **HR(95%CI)** | **P-value** |
| CD40, ng/mL | 113 | 1.12 (0.69, 1.83) | 0.6 |
| CD40 ligand ^*^, ng/mL | 107 | 1.10 (0.69, 1.75) | 0.6 |
| EN-RAGE ^*^, ng/mL | 115 | 0.97 (0.62, 1.54) | 0.9 |
| Eotaxin ^*^, pg/mL | 113 | 1.39 (0.91, 2.12) | 0.1 |
| FAS ^*^, ng/mL | 114 | 0.76 (0.48, 1.22) | 0.2 |
| HCC4, ng/mL | 115 | 0.93 (0.61, 1.41) | 0.7 |
| IL13 ^*^, pg/mL | 109 | 0.55 (0.34, 0.90) | **1.7 × 10^-2^** |
| IL16, pg/mL | 115 | 0.99 (0.67, 1.47) | 0.9 |
| IL17 ^*^, pg/mL | 106 | 0.89 (0.62, 1.30) | 0.5 |
| IL8 ^*^, pg/mL | 111 | 0.79 (0.51, 1.24) | 0.3 |
| MDC, pg/mL | 114 | 1.45 (0.97, 2.16) | 6.8 **×** 10^-2^ |
| MIP1 alpha ^*^, pg/mL | 114 | 1.02 (0.69, 1.49) | 0.9 |
| MIP1 beta ^*^, pg/mL | 114 | 0.66 (0.35, 1.24) | 0.1 |
| PARC, ng/mL | 115 | 0.82 (0.53, 1.27) | 0.3 |
| sRAGE ^*^, ng/mL | 112 | 0.92 (0.63, 1.34) | 0.6 |
| TRAILR3 ^*^, ng/mL | 114 | 0.69 (0.43, 1.10) | 0.1 |
| CFH ^*^, ug/mL | 109 | 1.00 (0.69, 1.44) | 0.9 |
| Complement 3^*^, mg/mL | 115 | 1.07 (0.73, 1.58) | 0.7 |
| IL18^*^, pg/mL | 114 | 1.11 (0.71, 1.76) | 0.6 |
| MCP1^*^, pg/mL | 115 | 0.92 (0.62, 1.35) | 0.6 |
| MIF^*^, ng/mL | 115 | 1.09 (0.68, 1.73) | 0.7 |
| RANTES^*^, ng/mL | 115 | 0.89 (0.59, 1.34) | 0.5 |
| Resistin^*^, ng/mL | 113 | 0.89 (0.59, 1.32) | 0.5 |
| TNFRII^*^, ng/mL | 115 | 0.77 (0.49, 1.21) | 0.2 |
| Il1ra^*^, pg/mL | 114 | 1.32 (0.86, 2.03) | 0.2 |
| CRP^*^, ug/mL | 110 | 1.05 (0.71, 1.54) | 0.8 |

Age and sex adjusted. The number of diabetics at baseline differs for each marker, after outliers exclusion. ^*^ Naturally log-transformed. CD40, cluster of differentiation 40; CD40 ligand, cluster of differentiation 40 ligand ; EN-RAGE, Extracellular Newly identified Receptor for Advanced Glycation End-products binding protein; FAS, Fas Cell Surface Death Receptor; HCC4, Human CC chemokine-4; IL13, interleukin 13; IL16, interleukin 16; IL17, interleukin 17; IL8, interleukin 8; MDC, Monocyte Derived Chemokine; MIP1alpha, Macrophage Inflammatory Protein 1 alpha; MIP1beta, Macrophage Inflammatory Protein 1 beta; PARC, Pulmonary and Activation-Regulated Chemokine; sRage, Soluble Receptor of Advanced Glycation End-products; TRAILR3, Tumor Necrosis Factor-related Apoptosis-inducing Ligand Receptor 3; CFH, Complement Factor H; IL18, interleukin 18; MCP1, Monocyte Chemotactic Protein 1; RANTES, Regulated Upon Activation, Normally T-Expressed, And Presumably Secreted; TNFR-II, Tumor Necrosis Factor Receptor 2; IL1ra, Interleukin 1 Receptor Antagonist; CRP, C-Reactive Protein. ^b^Sensitivity analysis: significant after Bonferroni correction (p = 0.05/26 = 1.9 × 10^-3^)
